# Evaluation of individualized para-tumor resection of cervical cancer patients based on three-dimensional reconstruction

**DOI:** 10.3389/fsurg.2023.1174490

**Published:** 2023-04-27

**Authors:** Lu Wang, Ping Liu, Hui Duan, Pengfei Li, Weili Li, Chunlin Chen

**Affiliations:** ^1^ Department of Obstetrics and Gynecology, Nanfang Hospital, Southern Medical University, Guangzhou, China; ^2^ Department of Obstetrics and Gynecology, The Second Affiliated Hospital of Zhengzhou University, Zhengzhou, China

**Keywords:** cervical cancer, para-tumor resection, tailoring surgery, three-dimensional reconstruction, stromal invasion

## Abstract

**Objective:**

To discuss the possibility of individualizing the para-tumor resection range (PRR) in cervical cancer patients based on three-dimensional (3D) reconstruction.

**Methods:**

We retrospectively included 374 cervical cancer patients who underwent abdominal radical hysterectomy. Preoperative computerized tomography (CT) or magnetic resonance imaging (MRI) data sets were collected to get 3D models. Postoperative specimens were measured to evaluate surgical scope. Oncological outcomes of patients with different depths of stromal invasion and PRR were compared.

**Results:**

A PRR of 32.35 mm was found to be the cut-off point. For the 171 patients with stromal invasion <1/2 depth, patients with a PRR over 32.35 mm had a lower risk of death and higher 5-year overall survival (OS) than that in the ≤32.35 mm group (HR = 0.110, 95% CI: 0.012–0.988, *P *= 0.046; OS: 98.8% vs. 86.8%, *P *= 0.012). No significant differences were found in 5-year disease-free survival (DFS) between the two groups (92.2% vs. 84.4%, *P *= 0.115). For the 178 cases with stromal invasion ≥1/2 depth, no significant differences were found in 5-year OS and DFS between groups (≤32.35 mm group vs. >32.35 mm group, OS: 71.0% vs. 83.0%, *P *= 0.504; DFS: 65.7% vs. 80.4%, *P *= 0.305).

**Conclusion:**

In patients with stromal invasion <1/2 depth, the PRR should reach 32.35 mm to get more survival benefit and in patients with stromal invasion ≥1/2 depth, the PRR should reach 32.35 mm at least to avoid worse prognosis. Cervical cancer patients with different depths of stromal invasion may receive tailoring resection of the cardinal ligament.

## 1. Introduction

Globally, especially in developing countries, cervical cancer still seriously threats the female reproductive health. In China, with the establishment of the national early cancer screening system and the advent of the vaccine era, the proportion of cervical cancer patients that could get early diagnosis and treatment has gradually increased, but the prevention and treatment of cervical cancer is still an arduous task (1–3). Surgical treatment is the main modality for early-stage cervical cancer, which achieved satisfactory 5-year survival rates. Minimal invasive approach to cervical cancer surgery has been debated after the publication of LACC trial. However, resent researchers have found that laparo-assisted vaginal radical hysterectomy does not affect survival rates of early-stage cervical cancer (4). Still, multiple clinical guidelines recommend abdominal type C radical hysterectomy (RH) plus pelvic lymphadenectomy as the standard surgical procedure for early-stage cervical cancer (5, 6). During type C RH, the cardinal ligaments (CL) are transected at pelvic side wall and the uterosacral ligaments (USL) are transected near sacral origin. However, the thorough removal of the CL and USL inevitably damages the pelvic autonomic nerves that run through. Our previous study has confirmed that more nerves within the CL are removed in RH than in nerve sparing radical hysterectomy (NSRH) (7). Consequently, the incidence of bladder, rectum, and sexual dysfunction remains high after surgery, which deeply troubled cervical cancer survivors who received RH.

The most common morbidity after RH for cervical cancer is urinary dysfunction, including hypocontractility of the bladder, incontinence, low bladder compliance, fistula and hydronephrosis (8). The long-term bladder function is significantly marked by reduction of bladder compliance, increase of residual urine volume, increase of first voiding desire and reduction of the maximum cystometric capacity (9, 10). A high incidence of urinary dysfunction occurred over 12 months after surgery (11), emphasizing the importance of standardized long-term follow-up in cervical cancer survivors. Sood et al. (12) reported 54% patients had intermittent flatus incontinence or constipation and no resolution were reported before 18-month visit. Sexual dysfunction, including anxiety, depression and sexual desire disorders were reported by over 50% patients and the symptoms were more severe over time (13–15). Moreover, the pattern and severity of surgical morbidity were strictly related to the extent of parametrial resection.

The key point of the conventional type C RH is the completeness and thoroughness of the surgery, including the total removal of the cervical ligaments, especially the CL. As for early-stage cancer, the surgery range should be confined to 1.0 cm–3.0 cm normal tissue around the tumor center. The concept of para-tumor resection is frequently mentioned in the treatment of multiple tumors. For instance, the intraoperative criterion for radical resection of liver cancer is that the margin should be over 1 cm (16). A retrospective multi-institution study in the Netherlands found that the extent of parametrectomy had no influence on survival for tumor of 20 mm or less and for larger tumors, a more radical hysterectomy might be associated with better DFS, indicating the possibly of individualized parametrectomy depending on tumor size (17). Therefore, we hypothesize that para-tumor resection might be applicable to cervical cancer so that for selected patients, individualized CL resection might help to limit the scope of surgery as much as possible while ensuring the survival.

For cervical cancer patients, para-tumor tissue includes the resection length of the ligaments and the distance between the tumor and the margin of the cervix. At present, limited methods of measuring, such as postoperative pathological measurement, are employed. However, digital three-dimensional (3D) reconstruction of preoperative CT or MRI data set could facilitate 3D view and accurate measurement of pelvic organ models. An individualized para-tumor resection might contribute to the reduction of surgical morbidity. Under this circumstance, this study aimed to explore a possible approach of individualized para-tumor resection for cervical cancer patients, by retrospectively evaluating the oncological outcomes of patients with different depths of stromal invasion and para-tumor resection range (PRR).

## 2. Materials and methods

### 2.1. Patients

**Figure 1:**
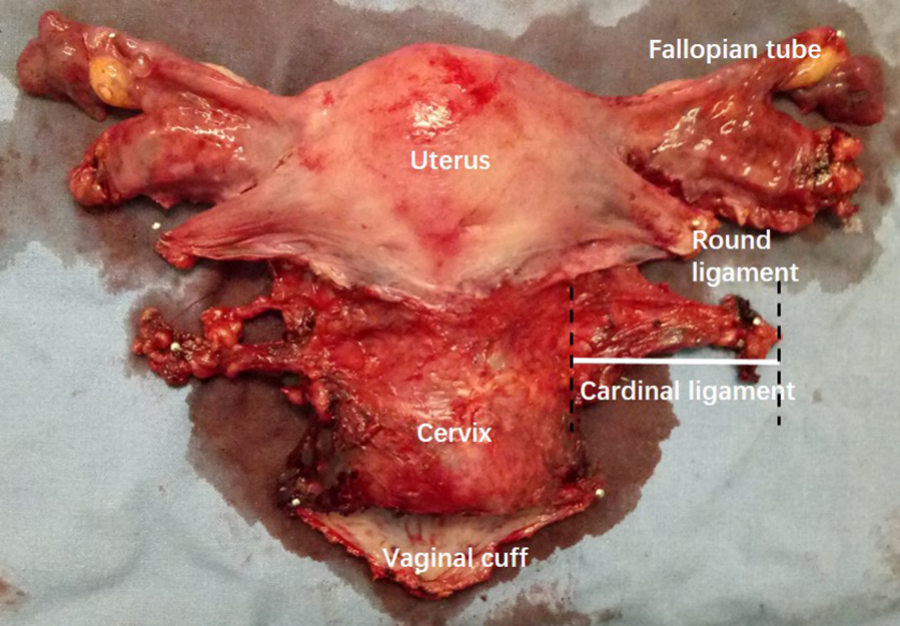
Measurement of the cardinal ligament (CL) on specimen. The length of the resected left CL was measured from the left or right cervical edge to the lateral edge of the taut surgical specimens.

We included 374 women patients with FIGO 2009 stage IB1 to IIA2 cervical cancer who were pathologically diagnosed by cervical biopsy at Nanfang Hospital, Southern Medical University, and underwent abdominal RH during February, 2010 to February 2018. The procedure of hysterectomy was conducted according to Querleu and Morrow's classification by Prof. Chunlin Chen (18). All patients were performed CT or MRI examination with thin-layer scans (<1 mm) preoperatively after consented. Exclusion criteria were as follows: (1) surgery other than type B or type C RH (including nerve-sparing procedures); (2) cervical conization before surgery; (3) history of radiotherapy or chemotherapy before surgery; (4) allergic to the contrast agent; (5) images unable to reconstruct or measure. After the uterus was removed during surgery, the specimens were measured. The length of the resected CL was measured from the left or right cervical edge to the lateral edge of the taut surgical specimens, respectively (Figure 1). The research protocol was approved by the Medical Ethics Committee of Nanfang Hospital (NFEC-2017-135), and written informed consent was obtained from all the participants.

### 2.2. Acquisition of datasets of CT or MRI

All CT data were collected from dual-source 64-slice spiral CT scans (SOMATOM Definition, Siemens, Germany). The patients were placed in the supine position for CT axial scanning and scanned from the 12th thoracic vertebra to 3 cm below the greater trochanter of the femur. An iodine-based contrast agent (Ultravist 370 mg/ml, Schering Pharmaceutical Ltd., Germany) was administered intravenously at 4 ml/s, followed by injection of 80 ml of normal saline. The setting of region of interest (ROI) and scan parameters were described in our previous study (19). The images were split into 1-mm slices and exported in the Digital Imaging and Communication in Medicine (DICOM) format for storage.

Static MRI scans were performed using a 3.0-T Philips Achieva TX scanner (Philips Medical Systems, Best, The Netherlands) with an eight-channel torso array coil. The patients were placed in the supine position and T2-weighted MRI images were acquired in the axial, sagittal and coronal planes at rest, as described in our previous study (20). The images were saved in the DICOM format on a disc.

### 2.3. 3D reconstruction and measurement of the pelvic organ models

All the image data in DICOM format were then imported into Mimics 10.01 (Materialize, Leuven, Belgium) software. The CL was reconstructed based on the CT venous phase and MRI T2W images. The method was based on our previous study and the deep uterine vein was used as the landmark for the recognition of the CL (19–21). The images were manually segmented according to the contours and then reconstructed in Mimics software using a surface-rendering algorithm. Other 3D models of the uterus, cervix, primary lesion, bladder, ureter, rectum, and uterine artery were reconstructed.

**Figure 2:**
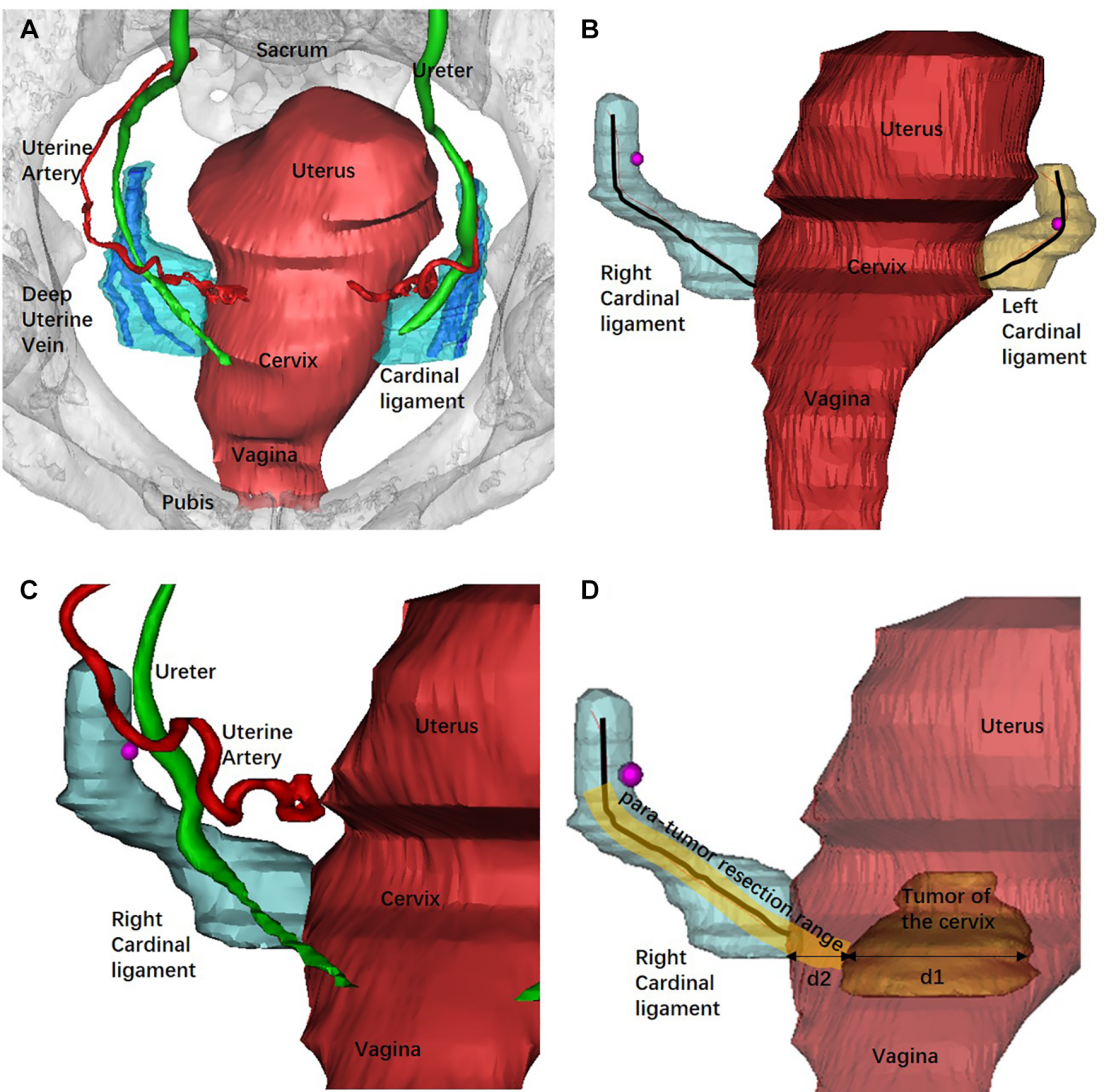
Reconstruction of the pelvic organs by Mimics software and the measurement of the 3D models. (**A**) The 3D model of pelvic and related organs from the superior aspect and the deep uterine vein was used as landmark to recognize the CL. (**B**) Bilateral CLs were reconstructed and measured using the fit centerline algorithm (black lines). (**C**) The intersection of the uterine artery and the ureter (rose red dot) was adopted as the landmark of the transection of CL in type B RH. (**D**) The measurement of the PRR. The tumor diameter was measured at the maximum cross sectional (*d*_1_). *d*_2_ represented the distance from the tumor border to the edge of the cervix. The PRR in type B RH was the sum of the resection length of the CL and *d*_2_ at the maximum cross sectional of the tumor diameter.

The 3D models were then exported as STL files into the same Mimics file to get an automatically matched model of the whole pelvis for measurement. The length of the CL was defined as the tissue around the deep uterine vein to the cervix or uterine corpus and was measured using the fit centerline algorithm by Mimics software. The CL removed during type B RH is approximately transected around the intersection of the uterine artery and the ureter. Thus, the length of the CL that should be removed during type B RH was measured as the distance from the cervix to the intersection of the uterine artery and the ureter in 3D models (*d*_in vivo_). The diameter of the tumor was measured at the maximum cross sectional. The tumor border to the edge of the cervix was defined as the distance from the lateral margin of the tumor to the lateral margin of the cervical stromal. We defined the 3D PRR as the sum of the conversional resection length of the CL and the distance from the tumor border to the edge of the cervix (Figure 2).

### 2.4. Statistical analysis

SPSS version 23.0 (IBM Corporation, Armonk, NY, USA) was used for statistical analysis. Intra-group correlation coefficient (ICC) was used for consistency test. Comparisons were performed *via* Student's *t*-test, matched samples *t*-test or ANOVA of repeated measurement data for continuous variables. X-tile software was used to get the best cut-off value. Overall survival (OS) was defined as the interval between surgery and the time of death due to any cause or the last valid follow-up. Disease-free survival (DFS) was defined as the interval between surgery and the time of disease progression/death or the last valid follow-up. Survival analysis was performed using the Kaplan-Meier method, log-rank test and Cox proportional hazards regression model with hazard ratios (HRs) and 95% confidence intervals (95% CIs). The difference was considered statistically significant when *P *< 0.05.

## 3. Results

Of the 374 consecutive patients (207 received type B RH and 167 received type C RH), 557 datasets were collected, including 354 CT datasets and 203 MRI datasets. The demographic and clinical characteristics of the patients are shown in Table 1. The average length of the resected left CL was 2.35 cm ± 0.83 cm. The average length of the resected right CL was 2.38 cm ± 0.91 cm. The average follow-up interval was 45.47 ± 4.22 months.

**Table 1:** Demographic data and clinical characteristics of the 374 included patients.

Characteristic	Value
Age (years), mean (SD)	50.08 (9.13)
Height (cm), mean (SD)	155.49 (7.30)
Weight (kg), mean (SD)	56.90 (8.94)
Body mass index (kg/m^2^), mean (SD)	23.40 (4.63)
The length of the resected left CL (cm), mean (SD)	2.35 (0.83)
The length of the resected right CL (cm), mean (SD)	2.38 (0.91)
Follow-up (months), mean (SD)	45.47 (4.22)
**FIGO stage, number (proportion)**	
IB1	145 (38.77%)
IB2	55 (14.71%)
IIA1	119 (31.82%)
IIA2	55 (14.71%)

Of all 374 patients, 186 underwent both simultaneous CT and MRI scans before surgery, and the consistency test of the 3D measurement was performed to avoid data error between the two datasets. The 3D measurements of the CL by CT and MRI scan datasets were 40.79 ± 9.67 mm and 45.68 ± 8.85 mm, respectively. In ICC analysis, a correlation coefficient of 0.715 was found (*P *< 0.001), which means the 3D measurement by the two different scan methods were in good consistent. On account of the better image forming of MRI, MRI image was given priority to get 3D reconstruction models in patients who underwent both examinations. For patients who only underwent CT scan before surgery, CT datasets were applied.

Among the 207 patients who received type B RH, 143 with best image development of the uterine artery and homolateral ureter in the preoperative CT scans were included in the establishment of the scale. The distance from the cervix to the intersection of the uterine artery and the ureter measured in 3D models (*d*_in vivo_) and the length of CL removed during surgery measured *in vitro* (*d*_in vitro_) were 25.18 ± 7.49 mm and 22.06 ± 6.61 mm, respectively. The difference between *d*_in vivo_ and *d*_in vitro_ was statistically significant (*t *= 5.098, *P *< 0.001). Ligaments *in vivo* might contract after removed from the body due to the change of physical mechanics and ischemia, so that a conversion scale should be established. The ratio between *d*_in vivo_ and *d*_in vitro_ was 1.25 ± 0.54 (average 1.25, median 1.13). We established two scales by the average and median ratio, respectively, to get the conversion length of the CL *in vitro*. The conversion lengths, *d_r_*_ = 1.25_ and *d_r_*_ = 1.13_, were compared with *d*_in vitro_ of the 143 patients in type B RH group to verify the accuracy of the scale. Statistically significant difference was found between the measurements by *d_r_*_ = 1.25_ and *d*_in vitro_ (20.14 ± 5.99 mm vs. 22.06 ± 6.61 mm, *t *= −3.497, *P *= 0.001). No difference was found between *d_r_*_ = 1.13_ and *d*_in vitro_ (22.28 ± 6.63 mm vs. 22.06 ± 6.61 mm, *t *= 0.394, *P *= 0.694). The conversion ratio of 1.13 had a better consistency. The conversion between *d*_in vivo_ and *d*_in vitro_ was eventually defined as *d*_in vitro_ equals to *d*_in vivo_/1.13. Thus, the PRR *in vivo* were converted as the following equation: PRR equals to the distance from the tumor border to the edge of the cervix plus the resection length of the CL *in vitro* multiply by 1.13.

**Figure 3:**
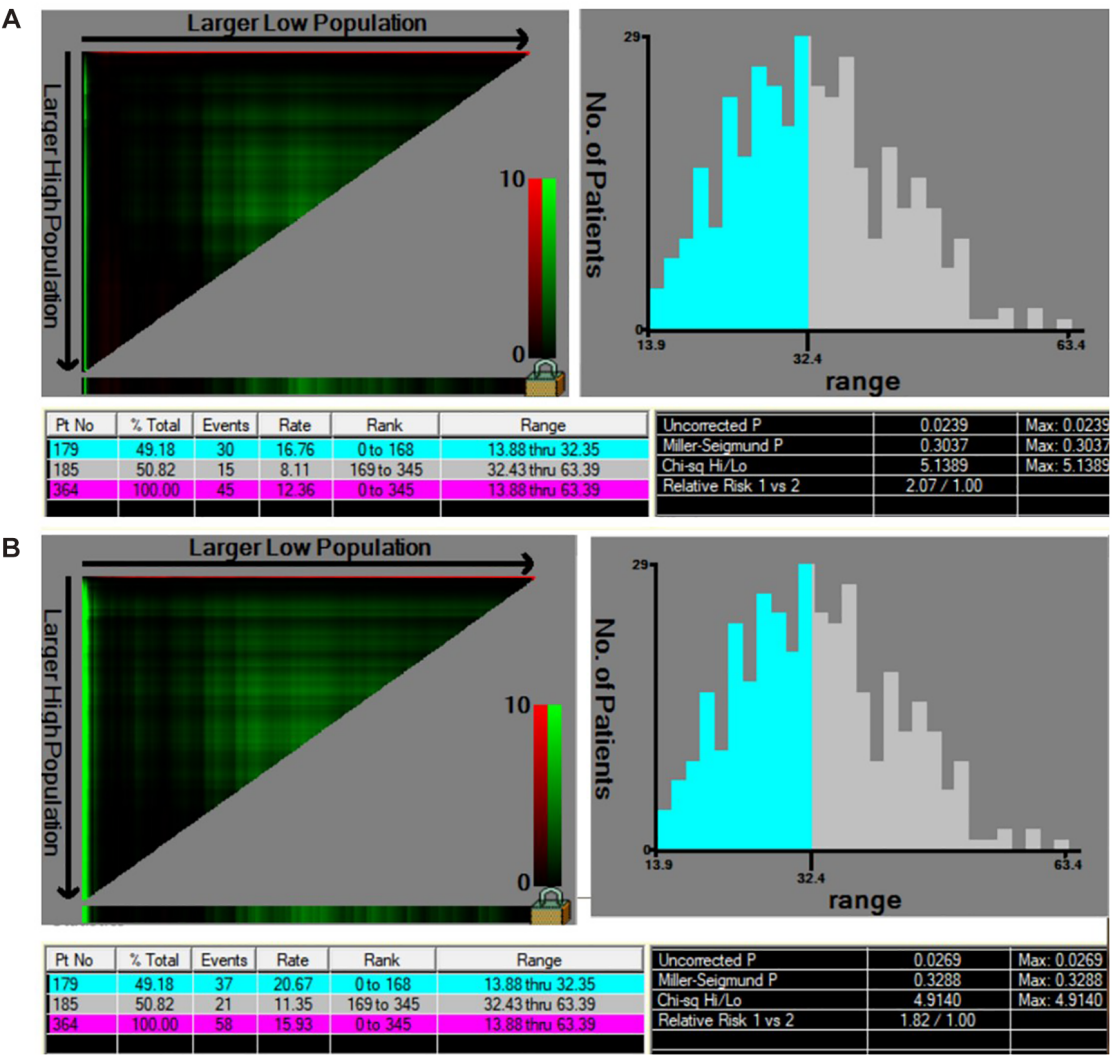
The cut-off value of PRR related to survival by X-tile. (**A**) The cut-off value of PRR considering OS was 32.35 mm (*χ*^2^ = 5.14, *P*_OS_*_ _*= 0.024). (**B**) The cut-off value of PRR considering DFS was 32.35 mm (*χ*^2^ = 4.91, *P*_DFS_*_ _*= 0.027).

**Figure 4:**
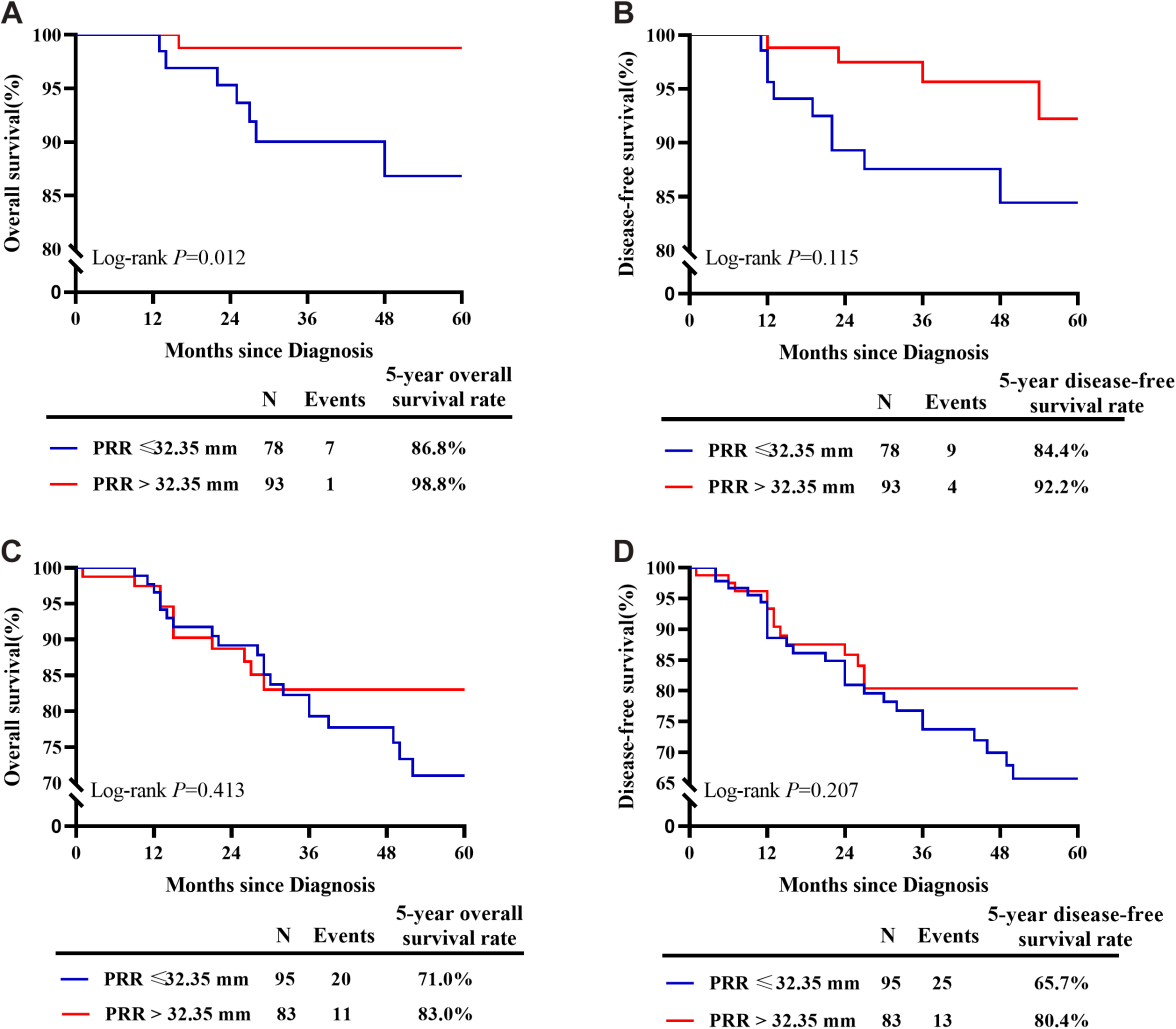
Survival curves of patients with PRR less than 32.35 mm and over 32.35 mm of different depths of stromal invasion. (**A**) In patients with stromal invasion <1/2 depth, patients with a PRR less than 32.35 mm showed less 5-year OS than those with a PRR over 32.35 mm. (**B**) In patients with stromal invasion <1/2 depth, no difference in 5-year DFS was found between patients with a PRR less than 32.35 mm and those with a PRR over 32.35 mm. (**C**) In patients with stromal invasion ≥1/2 depth, no difference in 5-year OS was found between patients with a PRR less than 32.35 mm and those with a PRR over 32.35 mm. (**D**) In patients with stromal invasion ≥1/2 depth, no difference in 5-year DFS was found between patients with a PRR less than 32.35 mm and those with a PRR over 32.35 mm.

Oncological outcomes of patients with different depth of cervical stromal invasion were then compared in all 374 patients. Excluding 10 patients in whom the resection lengths of para-tumor tissue was unable to evaluate, 364 patients were included in the final analysis. The PRR of the 364 patients was 35.83 ± 10.40 mm. The cut-off value of PRR related to survival was 32.35 mm (*P*_OS_*_ _*= 0.024, *P*_DFS_*_ _*= 0.027) (Figure 3). Fifteen patients were pathologically diagnosed with parametrial spread and were excluded. Of the 349 patients included in the analysis of survival, 171 were less than 1/2 depth stromal invasion and 178 were ≥1/2 depth stromal invasion. Survival analyses were conducted between patient with a PRR less than 32.35 mm and those with a PRR over 32.35 mm within groups of different stromal invasion. The clinical and pathological characteristics of the patients are presented in Table 2. In patients with stromal invasion <1/2 depth, patients with a PRR less than 32.35 mm showed less 5-year OS than those with a PRR over 32.35 mm (86.8% vs. 98.8%, *P *= 0.012; HR = 0.110, 95% CI: 0.012–0.988, *P *= 0.046). No difference in 5-year DFS was found between the two groups (84.4% vs. 92.2%, *P *= 0.115). In patients with stromal invasion ≥1/2 depth, no differences in 5-year OS and DFS were found between patients with a PRR less than 32.35 mm and those with a PRR over 32.35 mm (OS: 71.0% vs. 83.0%, *P *= 0.413, COX: *P *= 0.504; DFS: 65.7% vs. 80.4%, *P *= 0.207, COX: *P *= 0.305). Survival curves are shown in proportional hazards Figure 4 and COX analysis are shown in Table 3. In patients with stromal invasion <1/2 depth, the PRR should reach 32.35 mm to get more survival benefit and the resection length of CL in specimen should reach (32.35 mm minus the distance from the tumor border to the edge of the cervix)/1.13. In patients with stromal invasion ≥1/2 depth, the PRR should reach 32.35 mm at least to avoid worse prognosis and the resection length of CL in specimen should reach (32.35 mm minus the distance from the tumor border to the edge of the cervix)/1.13.

**Table 2:** Characteristics of the included cervical cancer patients with different depths of cervical stromal invasion.

	Less than 1/2 depth stromal invasion			≥1/2 depth stromal invasion		
	PRR less than 32.35 mm group (*n *= 78, %)	PRR over 32.35 mm group (*n* = 93, %)	*P* Value	PRR less than 32.35 mm group (*n* = 94, %)	PRR over 32.35 mm group (*n* = 81, %)	*P* Value
Age, year	50.94 ± 9.40	48.27 ± 9.10	0.063	49.55 ± 9.12	50.72 ± 8.49	0.384
Histological subtype	** **		0.932			0.339
Squamous-cell carcinoma	70 (89.70)	81 (87.10)		82 (86.32)	77 (92.77)	
Adenocarcinoma	6 (7.69)	8 (8.60)		10 (10.53)	6 (7.23)	
Adenosquamous carcinoma	1 (1.28)	2 (2.15)		1 (1.05)	0 (0)	
Other subtypes	1 (1.28)	2 (2.15)		2 (2.11)	0 (0)	
FIGO stage	** **		0.387			0.159
IB1	30 (38.46)	41 (44.09)		30 (31.58)	37 (44.58)	
IB2	9 (11.54)	16 (17.20)		13 (13.68)	11 (13.25)	
IIA1	28 (35.90)	23 (24.73)		37 (38.95)	20 (24.10)	
IIA2	11 (14.10)	13 (13.98)		15 (15.79)	25 (18.07)	
Tumor size	** **		0.068			0.080
≤4 cm	53 (67.95)	63 (67.74)		51 (53.68)	50 (60.24)	
>4 cm	22 (28.21)	18 (19.35)		43 (45.26)	28 (33.73)	
Unknown	3 (3.85)	12 (12.90)		1 (1.05)	5 (6.02)	
LVSI	** **		0.006			0.744
Negative	74 (94.87)	75 (80.65)		76 (80.00)	68 (81.93)	
Positive	4 (5.13)	18 (19.35)		19 (20.00)	15 (18.07)	
Vaginal margin	** **		0.358			0.283
Negative	78 (100.00)	92 (98.92)		95 (100.00)	82 (98.80)	
Positive	0 (0)	1 (1.08)		0 (0)	1 (1.20)	
Lymph node involvement	** **		0.258			0.401
Negative	63 (80.77)	81 (87.10)		68 (71.58)	64 (77.11)	
Positive	15 (19.23)	12 (12.90)		27 (28.42)	19 (22.89)	
Adjuvant therapy			0.795			0.051
None	33 (42.31)	36 (38.71)		29 (30.53)	38 (45.78)	
Chemotherapy	31 (39.74)	39 (41.94)		35 (36.84)	30 (36.14)	
Radiology	0 (0)	1 (1.08)		3 (3.16)	0 (0)	
Chemoradiotherapy	14 (17.95)	17 (18.28)		28 (29.47)	15 (18.07)	

**Table 3:** Cox regression of the included cervical cancer patients with different depths of cervical stromal invasion.

	Less than 1/2 depth stromal invasion		≥1/2 depth stromal invasion	
	OS	DFS	OS	DFS
	*P* Value (HR, 95% CI)	*P* Value (HR, 95% CI)	*P* Value (HR, 95% CI)	*P* Value (HR, 95% CI)
Histological subtype	0.030 (2.707, 1.099–6.666)	0.042 (1.937, 1.024–3.664)	0.808 (0.868, 0.279–2.704)	0.676 (0.816, 0.314–2.122)
FIGO stage	0.083 (2.347, 0.895–6.156)	0.858 (0.950, 0.539–1.674)	0.286 (1.190, 0.864–1.637)	0.101 (1.272, 0.954–1.697)
Tumor size	0.517 (0.567, 0.102–3.153)	0.325 (0.516, 0.139–1.924)	0.016 (2.359, 1.177–4.726)	0.076 (1.754, 0.943–3.264)
LVSI	0.181 (3.790, 0.537–26.723)	0.684 (0.709, 0.136–3.703)	0.046 (2.365, 1.015–5.510)	0.559 (1.262, 0.579–2.751)
Vaginal margin	0.993	0.989	0.990	0.983
Lymph node involvement	0.197 (2.951, 0.570–15.276)	0.097 (3.303, 0.805–13.550)	0.001 (9.158, 3.946–21.253)	0.001 (6.936, 3.341–14.402)
PRR	0.046 (0.110, 0.012–0.988)	0.115 (0.371, 0.180–1.273)	0.504 (0.771, 0.360–1.651)	0.305 (0.696, 0.349–1.390)
Adjuvant therapy	0.917 (1.042, 0.485–2.239)	0.096 (1.641, 0.916–2.940)	0.001 (0.566, 0.400–0.800)	0.034 (0.722, 0.534–0.976)

## 4. Discussion

Tailoring has become a major issue in cancer surgery. Attempts had been made to determine the adaptation of radicality to tumor spread in cervical cancer. Photopulos et al. recommended class II RH for cervical cancer patients with less than 3 mm invasion with possible invasion of lymphatic space, ≤3 mm invasion extending to the cone biopsy specimen and adenocarcinoma of uncertain invasion in their 1991 study (22). The adaptation was further extended to conization diagnosed exophytic squamous cell cervical cancer patients with less than 2 cm in diameter and less than 10 mm invasion, by Yang et al. (23). Landoni et al. conducted a prospective randomized study and found that, as a less radical surgery, class II RH had equally effect and a lesser degree of late complications compared with class III RH in surgical treatment of cervical cancer (24). Midterm analysis of a randomized controlled trial indicated that, in early early-stage cervical cancer patients with tumor diameter <2 cm and a depth of interstitial infiltrates <50%, type II RH had considerable security within 2 years after surgery and could improve the quality of life (25). We previously found that type B RH could be used for the treatment of stage IA1 to IIA2 cervical cancer (26). Despite the fine distinction in the scope, type II RH and type B RH might be feasible and safe in selected patients. For patients who want to preserve fertility, endocervicoscopy followed by conization might play a promising role (27). However, more tailored surgical scope considering individual tumor diameter and stromal invasion was not mentioned in the previous approach. Parametrectomy was considered the most significant procedure of cervical cancer surgery and the extensive resection of the CL is a must to ensure a disease-free state (28). In this study, we studied on the influence of individualizing PRR in cervical cancer patients with different tumor characteristics.

During type B RH, the ureter should be unroofed and mobilized laterally, permitting the transection of the para-cervix at the level of the ureteral tunnel. The ureter artery at the intersection of the uterine artery and the ureter could be the landmark (17, 21). In this study, we adopted this intersection as an anatomical landmark for the resection of the CL in type B RH, which facilitated the quantification of the *in vivo* length of the CL to be removed. However, we found a significant statistical difference between the *in vivo* and *in vitro* lengths of the CL (25.18 ± 7.49 mm vs. 22.06 ± 6.61 mm, *t *= 5.098, *P *< 0.001). Due to the change of physical mechanics and ischemia, the ligaments *in vivo* might contract after removed from the body, which result in the difference between *d*_in vivo_ and *d*_in vitro_. In order to further match the length of tissue, we established a conversion scale as *d*_in vitro_ equals to *d*_in vivo_/1.13.

Deep stromal invasion of the cervix is regarded as a risk factor for poor prognosis of cervical cancer patients (29). In cervical cancer patients with deep stromal invasion on one side of the cervix, the tumor may show a tendency of eccentric growth. Bilateral cardinal ligaments resection in these patients might be individualized to minimize nerve trauma, as experiments has confirmed multiple nerve types are present within the CL (7, 30). To our knowledge, data on the individualized excision of the CL in cervical cancer patients are scarce. We attempt to find a quantitative index for cervical cancer patients to achieve the goal of minimum trauma under maximum prognosis. In this study, we only took into account the deeper side of stromal invasion in patients with different depths of stromal invasion on two sides. To accurate the resection ranges of para-tumor tissue, we defined PRR as the sum of the resection length of the CL and the distance from the tumor border to the edge of the cervix. Combined with the follow-up data and using X-tile software, we obtained the possible cutoff value of the PRR related to OS and DFS as 32.35 mm, and further analyzed the differences in the oncology outcomes of different PRRs in patients with different depths of stromal invasion. The results showed that patients with cervical cancer with superficial 1/2 stromal invasion could benefit more in 5-year OS and DFS when the PRR was more than 32.35 mm, while for patients with deep 1/2 stromal invasion, the PRR should be at least 32.35 mm to ensure the 5-year OS and DFS. The individualization of PRR we found is basically depends on the tumor size and the depth of stomal invasion. It seems that patients with superficial 1/2 stromal invasion have a larger resection range. But in fact, the distance from the tumor edge to the cervical edge is often smaller in patients with deep stromal invasion, and much larger in patients with superficial stromal invasion, thus the result is reasonable.

## 5. Conclusions

In patients with stromal invasion <1/2 depth, the PRR should reach 32.35 mm to get more survival benefit and in patients with stromal invasion ≥1/2 depth, the PRR should reach 32.35 mm at least to avoid worse prognosis. Cervical cancer patients with different depths of stromal invasion may receive tailoring resection of the cardinal ligament.

## Data Availability

The raw data supporting the conclusions of this article will be made available by the authors, without undue reservation.

## References

[1] Chen W Zheng R Baade PD Zhang S Zeng H Bray F Cancer statistics in China, 2015. CA Cancer J Clin. (2016) 66(2):115–32. 10.3322/caac.21338 26808342

[2] Feng RM Zong YN Cao SM Xu RH Current cancer situation in chinia: good or bad news from the 2018 global cancer statistics? Cancer Commun. (2019) 39(1):22. 10.1186/s40880-019-0368-6 PMC6487510 31030667

[3] Di J Rutherford S Chu C. Review of the cervical cancer burden and population-based cervical cancer screening in China. Asian Pac J Cancer Prev. (2015) 16(17):7401–7. 10.7314/apjcp.2015.16.17.7401 26625735

[4] Ronsini C Köhler C De Franciscis P La Verde M Mosca L Solazzo MC Laparo-assisted vaginal radical hysterectomy as a safe option for minimal invasive surgery in early stage cervical cancer: a systematic review and meta-analysis. Gynecol Oncol. (2022) 166(1):188–95. 10.1016/j.ygyno.2022.04.010 35513934

[5] Bhatla N Aoki D Sharma DN Sankaranarayanan R. Cancer of the cervix uteri: 2021 update. Int J Gynaecol Obstet. (2021) 155(Suppl 1):28–44. 10.1002/ijgo.13865 34669203 PMC9298213

[6] Abu-Rustum NR Yashar CM Bean S Bradley K Campos SM Chon HS NCCN Guidelines insights: cervical cancer, version 1.2020. J Natl Compr Canc Netw. (2020) 18(6):660–6. 10.6004/jnccn.2020.0027 32502976

[7] Chen C Li W Li F Liu P Zhou J Lu L Classical and nerve-sparing radical hysterectomy: an evaluation of the nerve trauma in cardinal ligament. Gynecol Oncol. (2012) 125(1):245–51. 10.1016/j.ygyno.2011.12.448 22209773

[8] Wit EMK Horenblas S. Urological complications after treatment of cervical cancer. Nat Rev Urol. (2014) 11:110–7. 10.1038/nrurol.2013.323 24473416

[9] Laterza RM Sievert KD de Ridder D Vierhout ME Haab F Cardozo L Bladder function after radical hysterectomy for cervical cancer. Neurourol Urodyn. (2015) 34(4):309–15. 10.1002/nau.22570 24519734

[10] Cao TT Wen HW Gao YN Lyu QB Liu HX Wang S Urodynamic assessment of bladder storage function after radical hysterectomy for cervical cancer. Chin Med J (Engl). (2020) 133(19):2274–80. 10.1097/CM9.0000000000001014 32925291 PMC7546844

[11] Plotti F Angioli R Zullo MA Sansone M Altavilla T Antonelli E Update on urodynamic bladder dysfunctions after radical hysterectomy for cervical cancer. Crit Rev Oncol Hematol. (2011) 80(2):323–9. 10.1016/j.critrevonc.2010.12.004 21277788

[12] Sood AK Nygaard I Shahin MS Sorosky JI Lutgendorf SK Rao SS. Anorectal dysfunction after surgical treatment for cervical cancer. J Am Coll Surg. (2002) 195(4):513–9. 10.1016/s1072-7515(02)01311-x 12375757

[13] Pieterse QD Kenter GG Maas CP de Kroon CD Creutzberg CL Trimbos JB Self-reported sexual, bowel and bladder function in cervical cancer patients following different treatment modalities: longitudinal prospective cohort study. Int J Gynecol Cancer. (2013) 23(9):1717–25. 10.1097/IGC.0b013e3182a80a65 24172106

[14] Wallin E Falconer H Rådestad AF. Sexual, bladder, bowel and ovarian function 1 year after robot-assisted radical hysterectomy for early-stage cervical cancer. Acta Obstet Gynecol Scand. (2019) 98(11):1404–12. 10.1111/aogs.13680 31237957

[15] Wang X Chen C Liu P Li W Wang L Liu Y. The morbidity of sexual dysfunction of 125 Chinese women following different types of radical hysterectomy for gynaecological malignancies. Arch Gynecol Obstet. (2018) 297:459–66. 10.1007/s00404-017-4625-0 29282516

[16] Zhou J Sun H Wang Z Cong W Wang J Zeng M Guidelines for the diagnosis and treatment of hepatocellular carcinoma (2019 edition). Liver Cancer. (2020) 9(6):682–720. 10.1159/000509424 33442540 PMC7768108

[17] Derks M van der Velden J de Kroon CD Nijman HW van Lonkhuijzen LRCW van der Zee AGJ Surgical treatment of early-stage cervical cancer: a multi-institution experience in 2124 cases in The Netherlands over a 30-year period. Int J Gynecol Cancer. (2018) 28(4):757–63. 10.1097/IGC.0000000000001228 29595758

[18] Querleu D Morrow CP. Classification of radical hysterectomy. Lancet Oncol. (2008) 9(3):297–303. 10.1016/S1470-2045(08)70074-3 18308255

[19] Duan H Liu P Chen C Chen L Li P Li W Reconstruction of three-dimensional vascular models for lymphadenectomy before surgery. Minim Invasive Ther Allied Technol. (2020) 29(1):42–8. 10.1080/13645706.2019.1569533 30794060

[20] Chen J Chen C Li Y Chen L Xu J Liu P Impact of radical hysterectomy on the transobturator sling pathway: a retrospective three-dimensional magnetic resonance imaging study. Int Urogynecol J. (2018) 29(9):1359–66. 10.1007/s00192-017-3533-y 29242958

[21] Cibula D Abu-Rustum NR Benedetti-Panici P Köhler C Raspagliesi F Querleu D New classification system of radical hysterectomy–emphasis on a three-dimensional anatomic template for parametrial resection. Gynecol Oncol. (2011) 122(2):264–8. 10.1016/j.ygyno.2011.04.029 21592548

[22] Photopulos GJ Zwaag RV. Class II radical hysterectomy shows less morbidity and good treatment efficacy compared to class III. Gynecol Oncol. (1991) 40(1):21–4. 10.1016/0090-8258(91)90078-j 1989910

[23] Yang YC Chang CL. Modified radical hysterectomy for early ib cervical cancer. Gynecol Oncol. (1999) 74(2):241–4. 10.1006/gyno.1999.5434 10419738

[24] Landoni F Maneo A Cormio G Perego P Milani R Caruso O Class II versus class III radical hysterectomy in stage IB-IIA cervical cancer: a prospective randomized study. Gynecol Oncol. (2001) 80(1):3–12. 10.1006/gyno.2000.6010 11136561

[25] Sun H Cao D Shen K Yang J Xiang Y Feng F Piver type II vs. Type III hysterectomy in the treatment of early-stage cervical cancer, midterm follow-up results of a randomized controlled trial. Front Oncol. (2018) 8:568. 10.3389/fonc.2018.00568 30555800 PMC6280766

[26] Chen C Wang W Liu P Li P Wang L Jin S Survival after abdominal Q-M type B versus C2 radical hysterectomy for early-stage cervical cancer. Cancer Manag Res. (2019) 11:10909–19. 10.2147/CMAR.S220212 32021416 PMC6955639

[27] Della Corte L Mercorio A Giampaolino P Vitale SG Vizzielli G Bifulco G The role of endocervicoscopy in women with cervical intraepithelial neoplasia: a systematic review of the literature. Updates Surg. (2022) 74(4):1239–45. 10.1007/s13304-021-01193-2 34739708

[28] Clark KM Pandya AM. Abdomen and Pelvis: Cardinal Ligaments (Mackenrodts, Transverse Cervical, or Lateral Cervical Ligaments) [Updated 2022 Sep 19]. In: StatPearls [Internet]. Treasure island (FL): StatPearls Publishing (2023). Available from: https://www.ncbi.nlm.nih.gov/books/NBK557384/#_NBK557384_pubdet_. 32491316

[29] Sedlis A Bundy BN Rotman MZ Lentz SS Muderspach LI Zaino RJ A randomized trial of pelvic radiation therapy versus no further therapy in selected patients with stage IB carcinoma of the cervix after radical hysterectomy and pelvic lymphadenectomy: a gynecologic oncology group study. Gynecol Oncol. (1999) 73(2):177–83. 10.1006/gyno.1999.5387 10329031

[30] Butler-Manuel SA Buttery LD A'Hern RP Polak JM Barton DP Pelvic nerve plexus trauma at radical and simple hysterectomy: a quantitative study of nerve types in the uterine supporting ligaments. J Soc Gynecol Investig. (2002) 9(1):47–56. 10.1016/s1071-5576(01)00145-9 11839509

